# Case of Hydrops Pericardii Suddenly Formed, with Remarks

**Published:** 1847-05

**Authors:** 


*Case of hydrops Pericardii suddenly formed, with Remarks*, by S. Jackson, M. D., of Philadelphia, formerly of Northumberland.

In the summer of 1845, I was hurried to a Mrs. C., who was said to be in a dying state. Dr. Bryan, whom I found at the bedside, had been there a few minutes and had been judiciously employed in fanning the remaining embers. While, with forlorn hope, I was writing a prescription, she gently breathed her last. The sad catastrophe was hardly over when Dr. S. G. Morton, her accoucheur, arrived and gave us the following history of the case.

About six hours previous to this, she had been delivered of her first child, after a severe labour, which she bore with fortitude, and went through without any unpleasant symptoms. The placenta did not come away by the natural efforts ; and, upon introducing the hand, after waiting several hours, there was found an hour-glass contraction, to overcome which he was obliged to use considerable exertion. After this she appeared very well, complained only of drowsiness and some little debility ; but the pulse was regular and the Dr. left her with entire confidence, after prescribing food and rest.

Her mother stated that about an hour after the Dr. had retired, they became alarmed at her paleness and debility, upon which they sent for him, in haste, but he was not at home. The womb was firmly contracted and there had been no hæmorrhage. Here then, was an inextricable history which nothing but necrotomy could explain, and this was made by Professor Goddard the following day.

The uterus was well contracted and perfectly normal, the abdomen and its viscera entirely sound, the cut muscles looked very pale, *the pericardium contained at least two quarts of water, almost limpid*, no inflammation in this organ, nothing abnormal in the whole chest except the water.

This woman had been, as every one supposed, perfectly well till she began to look pale and to complain of sinking, about an hour and a half or two hours after the delivery of the placenta. There was no water in the pleuræ or abdomen; none in the brain, as her mind was clear to the last; there was no anasarca, not even of the feet. The effusion could not have existed in any appreciable degree in the morning, for she had walked up a long staircase and had gone through a painful labour without any anhelation. She could not have been panting with a dydrops pericardii during her hard labour without its having been noticed by her careful accoucheur. Her labour lasted about eight hours; the placenta was taken away about three hours after her delivery of the child; and, within three more she was dead,—a tremendous catastrophe to which nothing could reconcile us poor anxious practitioners of the healing art except the fortunate discovery of the cause. But the cause of this cause is still a most important desideratum. Dr. Darwin might refer it to his retroverted lymphatics, but this hypothesis requires another, this a third, and so on without end, in the manner of those who have been in the practice of theorizing on the causes of fever.

This case appears to be analogous in its sudden formation to those suddenly formed hydroceles of which an example was recorded in the preceding number of this Journal by Dr. H. H. Smith.

A similar case has also been related by myself in the number of this journal for January, 1840, and as the number is out of print, and may not be in the hands of all the present subscribers, I shall here quote the case as there related.

"Case.—In the winter of 1829, I was requested by Dr. Price of Sunbury, to consult with him in the case of Michael Hoffman, supposed to be labouring under strangulated hernia. The Doctor had tried the taxis freely, and we both tried it again, having prepared the system by the tobacco enemata. This having failed, we agreed to meet in the afternoon and to bring Dr. Rodrigue to assist at the operation, should it prove necessary.

"We met at the appointed hour and agreed that the operation should be immediately performed. Upon making the usual incision, about six inches long, I was soon surprised with the appearance of a sac, smooth and tense, as though filled with water alone. Upon placing it between my eyes and the setting sun, which shone brilliantly upon us, I saw the whole contents of the sac were uniform and semi-transparent. I said, *tacete, tacete—hydrocele est*, and immediately plunged the scalpel into the tumour and discharged the water by a large incision. I manipulated and conducted the affair in such a manner, that neither the bystanders nor the patient suspected anything extraordinary. I drew the upper end of the wound together by two stiches and covered the whole with a poultice of bread and milk. He had an easy, rapid recovery, and the hydrocele was radically cured.

"The deception and erroneous diagnosis were not more extraordinary than the history of the case. The man had told Dr. Price precisely as he afterwards told us both, that the whole tumour had suddenly come on that morning upon his lifting a heavy log; and, further, that he had never known it before. Having heard its history from Dr. Price, I made no examination, taking it for hernia on the report of a skilful physician. The tumor was very large, and gave us the idea of a hernia containing much omentum."

The above patient was a man of good character and rational mind, and surely he had no motive to falsify. I have said, above, that "the whole tumour had suddenly came on that morning upon lifting a heavy log;" this was reported to me both by the patient and his physician: but I have to blame myself here for an important deficit—I no not remember making any inquiry, whether the tumefaction was absolutely sudden in the precise manner of a rupture, or whether it was gradual through the space of an hour or two. It could not have been long in forming, for the man had gone out to his day's labour in a short winter's morning; had brought on the disease; had sent two miles for Dr. Price; the Doctor had done all that he could venture to do by the attempted taxis; and, had sent for me more than two miles—all which was done long before midday. The patient's own opinion was that he was ruptured.—*Am. Jour. Med. Sciences.*

